# IDH1/2 Mutations in Cancer Stem Cells and Their Implications for Differentiation Therapy

**DOI:** 10.1369/00221554211062499

**Published:** 2021-12-30

**Authors:** Remco J. Molenaar, Johanna W. Wilmink

**Affiliations:** Department of Hematology, Cancer Center Amsterdam, Amsterdam University Medical Centers, Amsterdam, The Netherlands; Department of Medical Oncology, Cancer Center Amsterdam, Amsterdam University Medical Centers, Amsterdam, The Netherlands; Department of Medical Oncology, Cancer Center Amsterdam, Amsterdam University Medical Centers, Amsterdam, The Netherlands

**Keywords:** cancer stem cells, chemotherapy, differentiation, enasidenib, isocitrate dehydrogenase, ivosidenib, targeted therapy, therapy responses, 2-hydroxyglutarate

## Abstract

Isocitrate dehydrogenase 1 and 2 (IDH1/2) are enzymes recurrently mutated in various types of cancer, including glioma, cholangiocarcinoma, chondrosarcoma, and acute myeloid leukemia. Mutant IDH1/2 induce a block in differentiation and thereby contribute to the stemness and oncogenesis of their cells of origin. Recently, small-molecule inhibitors of mutant IDH1/2 have been Food and Drug Administration–approved for the treatment of *IDH1/2*-mutated acute myeloid leukemia. These inhibitors decrease the stemness of the targeted *IDH1/2*-mutated cancer cells and induce their differentiation to more mature cells. In this review, we elucidate the mechanisms by which mutant IDH1/2 induce a block in differentiation and the biological and clinical effects of the release into differentiation by mutant-IDH1/2 inhibitors. **(J Histochem Cytochem 70:83–97, 2022)**

## Introduction

Cancer stem-like cells (CSCs) are malignant cells with stem cell characteristics, specifically the potential for self-renewal and differentiation. Similar to stem cells in benign tissues that support the renewal of more mature cells, CSCs are hypothesized to replace malignant cells with a more limited lifespan and therefore enable the persistence of cancer in the presence or absence of cancer therapy and the metastasis of tumors. The CSC hypothesis is based on experimental models with immunodeficient mice in whom a small subset of cells from human tumors can be engrafted and populate the full diversity of malignant cells that were present in the original tumor, whereas other subsets of cancer cells do not have this ability. Although this definition of CSCs is thus largely based on the ability to engraft a tumor in an experimental mouse model and not necessarily clinical observations in human patients, the development of novel cancer therapies is increasingly impacted by the rationale that deep and durable remissions can best be achieved by targeting the CSCs and not just the tumor bulk.^1^

Isocitrate dehydrogenase 1 and 2 (IDH1/2) are enzymes that perform key roles in various cellular functions, including the regulation of carbohydrate metabolism, epigenetics, differentiation, DNA repair, and redox states. Wild-type IDH1 and IDH2 (wtIDH1/2) oxidize and decarboxylate isocitrate to α-ketoglutarate (α-KG) in the cytoplasm and mitochondria, respectively, and simultaneously reduce NADP^+^ to NADPH. Through these metabolites, wtIDH1/2 function in the aforementioned myriad of cellular processes because α-KG is a core metabolite of the tricarboxylic acid (TCA) cycle and is also necessary for the function of α-KG-dependent dioxygenases that are involved in epigenetic regulation and DNA repair.^2^ In addition, NADPH is one of the most important sources of reducing power in most human tissues and is therefore essential in maintaining cellular redox states.^3,4^ *IDH1/2* mutations (*IDH1/2*mt) are almost always heterozygous and occur in a hotspot fashion in the enzymatically active sites, for example, *IDH1*^R132^, *IDH2*^R140^, and *IDH2*^R172^, to enable a neomorphic reaction by mutant IDH1/2 (mtIDH1/2) that converts α-KG into *D*-2-hydroxyglutarate (*D*-2-HG).^5^ Human cells have a very limited capacity to metabolize *D*-2-HG, which subsequently accumulates to millimolar concentrations and competitively inhibits α-KG-dependent dioxygenases.^5–9^ Therefore, *D*-2-HG is considered to be an oncometabolite. In addition, *IDH1/2*mt cancer cells have a decreased NADPH production capacity because mtIDH1/2 enzymes have lost the ability to reduce NADP^+^ to NADPH but instead oxidize NADPH to NADP^+^ to catalyze the conversion from α-KG to *D*-2-HG.^4,5,10^ Together, the *D*-2-HG accumulation and the decreased NADPH production capacity affect the abovementioned plethora of cellular functions, which may all contribute to oncogenesis and have been extensively reviewed before.^2,9,11^

*IDH1/2*mt are found in multiple cancer types, such as glioma and glioblastoma,^10,12,13^ chondrosarcoma,^14^ cholangiocarcinoma,^15^ myelodysplastic syndrome (MDS), and acute myeloid leukemia (AML).^16–18^ In the origin of these types of cancer, *IDH1/2*mt are considered to be inaugural or at least early events.^14,19–22^ In the case of MDS and AML, the data are conflicting because it has been postulated that *IDH1/2*mt are not necessarily early events in the formation of AML, but rather drive progression from precursor states such as MDS to full-blown AML.^18^ In tumors where *IDH1/2*mt are indeed inaugural or early genetic events, they are present in all, or at least the large majority of, cancer cells including the CSCs.^22^ As a consequence, *IDH1/2*mt that occur as early genetic events are important targets for cancer therapy because then all subclones contain the *IDH1/2*mt and are sensitive to mtIDH1/2 inhibitors. This appreciation of the contribution of *IDH1/2*mt to oncogenesis motivated the development of mtIDH1/2 inhibitors.^23^ These small-molecule inhibitors effectively reduce the production of *D*-2-HG by mtIDH1/2 and are also known as sidenibs.^24,25^ Examples include the mtIDH1 inhibitor ivosidenib, which is Food and Drug Administration (FDA)-approved for the treatment of *IDH1*mt newly diagnosed (ND), refractory or relapsed (R/R) AML and *IDH1*mt previously treated, locally advanced, or metastatic cholangiocarcinoma^26–29^; the mtIDH2 inhibitor enasidenib, which is FDA-approved for the treatment of *IDH2*mt R/R AML^30,31^; the dual mtIDH1/2 inhibitor vorasidenib, which is being investigated for *IDH1/2*mt recurrent or progressive glioma^32^; and various other mtIDH1/2 inhibitors that are still in clinical trials.^33^ In addition to the aforementioned indications, ivosidenib is currently being investigated for the treatment of *IDH1*mt glioma and chondrosarcoma in early-phase clinical trials^34,35^ and enasidenib is studied in *IDH2*mt MDS.^36^ Most clinical trials were conducted with mtIDH1/2 inhibitors as monotherapy, but ivosidenib and enasidenib have also been investigated in combination with intensive chemotherapy or azacitidine for AML.^37–39^

## *IDH1/2*mt in Cancer Cell Stemness and Differentiation

During the early preclinical investigation of the biological effects of mtIDH1/2 and their inhibitors, the increased stemness of *IDH1/2*mt cancer cells and its underlying mechanisms were already appreciated. First, several hallmarks of cellular dedifferentiation were observed when *IDH1/*2mt were introduced into in vitro and in vivo experimental models. Second, preclinical, translational, and clinical studies with mtIDH1/2 inhibitors consistently showed that these agents reverse this stemness and promote cellular differentiation of *IDH1/2*mt cancer cells. Both will be discussed in this review.

### Epigenetic Hypermethylation and Cellular Dedifferentiation in mtIDH1/2 Cells

DNA and histone hypermethylation is a feature that is observed across most investigated types of cancer that frequently contain *IDH1/2*mt, such as glioma,^40^ chondrosarcoma,^41,42^ cholangiocarcinoma,^20^ and AML.^16^ Furthermore, genomic hypermethylation and an associated block of differentiation are induced by the expression of mtIDH1/2 or the administration of *D*-2-HG in cell models related to these types of cancer, such as primary human astrocytes,^43,44^ human and mouse neural stem cells,^45–47^ human and mouse mesenchymal stem cells,^42,48^ mouse hepatoblasts,^49^ and mouse hematopoietic cells,^16,50,51^ but also human embryonic cells and mouse adipocytes.^52^ These findings were associated with an increased number of *IDH1/*2mt cancerous stem cells when these mutations were induced in benign stem or progenitor cells of these tissues.^16,42,44,46–50,52,53^ DNA and histone hypermethylation occurs because the accumulated *D*-2-HG in *IDH1/2*mt cells inhibits α-KG-dependent demethylases, such as the DNA demethylase TET2 and the family of histone lysine demethylases (KDMs).^6,16,43,52^ The need for *IDH1/2*mt cancer cells to induce epigenetic hypermethylation and suppress TET2 function is emphasized by the finding that in several *IDH1/2*mt AML patients who were successfully treated with an mtIDH1/2 inhibitor but whose AML subsequently relapsed, a novel *TET2* mutation that was not present at baseline emerged and DNA hypermethylation was sustained despite low *D*-2-HG levels.^54^ In addition to epigenetic mechanisms, *IDH1/2*mt also blocks differentiation via metabolic pathways. For example, mtIDH1 inhibits α-KG-dependent dioxygenases necessary for succinyl-CoA production, a critical component for heme production. The resulting succinyl-CoA deficiency attenuates heme biosynthesis in *IDH1*mt hematopoietic cells, blocking erythroid differentiation at the late erythroblast stage and the erythroid commitment of hematopoietic stem cells.^51^

### Induction of Differentiation by mtIDH1/2 Inhibitors in Preclinical Models

In preclinical mechanistic studies, inhibition of mtIDH1 in primary *IDH1*mt glioma xenografts grown in mice induced the expression of genes associated with both astrocytic and oligodendrocytic differentiation (*GFAP*, *AQP4*, *ATP1A2*, *PTGDS*, and *ZBTB16*), and this effect was associated with reduced repressive H3K9 and H3K27 trimethylation on the promoters of these genes.^24,55,56^ In addition, mtIDH1/2 inhibition in *IDH1/2*mt AML cells induces differentiation. This was observed in an *IDH2*mt erythroleukemia cell line, in which erythropoietin (EPO)-induced differentiation to red blood cells was restored by mtIDH2 inhibition,^25,57,58^ and this was associated by the reversion of DNA and histone hypermethylation.^58^ Crucially, treatment of these *IDH2*mt erythroleukemia cells with an mtIDH2 inhibitor did not induce apoptosis even at high dosages, suggesting that the antileukemic efficacy of mtIDH2 inhibitors is not based on cytotoxicity but on releasing the brakes on differentiation of *IDH2*mt AML cells that are trapped in the stem and progenitor cell compartment.^57^ Similar findings were seen in primary human *IDH1*mt or *IDH2*mt AML cells in vitro and in mice xenografts, in which mtIDH1 or mtIDH2 inhibition induced blast differentiation as shown by an increase in cells that were positive for cell surface markers associated with monocytic and granulocytic differentiation (CD11b, CD14, CD15) and intracellular myeloperoxidase (MPO) as marker of maturation into the neutrophilic pathway.^25,57,59,60^ Quantitatively, 20 days of mtIDH2 inhibition in primary human *IDH2*mt AML mouse xenografts induced differentiation of 70% of the human blood cells into the monocytic/macrophage and granulocytic lineages, which was associated with a mild to marked decrease in the percentage of human blasts (2- to 35-fold).^57^ Furthermore, functional assays during mtIDH2 inhibition in primary human *IDH2*mt AML cells in vitro yielded the presence of mature, functional neutrophils with phagocytic activity.^57^ Differentiation induction of primary human *IDH1*mt AML cells by mtIDH1 inhibition was associated with reduced histone trimethylation levels at the H3K4, H3K9, H3K27, and H3K36 loci, as well as reduced global DNA methylation.^59,60^

However, not all epigenetic effects of *IDH1/2*mt are reversible. In primary *IDH1*mt glioma xenografts grown in mice, an mtIDH1 inhibitor induced demethylation of histone markers (see above) without appreciable changes in genome-wide DNA methylation.^24^ Furthermore, in some preclinical glioma models, mtIDH1 inhibition had no effect on tumor growth, histone or genome-wide DNA methylation, or the expression of genes associated with stemness or glial differentiation.^61^ In addition, long-term studies on inducible *IDH1*mt in immortalized human astrocytes revealed that mtIDH1 results in progressive and sometimes irreversible accumulation of methylation marks on DNA and histones because the epigenome and transcriptome do not completely return to the original state after the subsequent long-term discontinuation of mtIDH1 expression. In agreement with these findings, mouse xenografts continuously expressing mtIDH1 exhibited a tumor growth rate that was comparable to mouse xenografts with discontinued expression of mtIDH1. On the molecular level, *L1CAM*, a marker for glioma stem cells, was among the genes that was persistently upregulated despite long-term loss of mtIDH1 expression, and L1CAM is associated with gliomagenesis in xenograft models.^44^ In clinical studies with mtIDH1/2 inhibitors in *IDH1/2*mt AML patients, potent suppression of *D*-2-HG is sometimes observed without epigenetic demethylation. This phenomenon was associated with primary resistance against mtIDH1/2 inhibitors.^54^

### Induction of Differentiation by mtIDH1/2 Inhibitors in Translational Studies

In support of these preclinical findings, induction of differentiation has also been shown in patients with *IDH1/2*mt malignancies that were treated with mtIDH1/2 inhibitors. Translationally, the induction of differentiation has been most thoroughly studied in the context of enasidenib treatment for *IDH2*mt R/R AML. Direct and morphologic evidence of myeloid differentiation during enasidenib treatment was shown in a patient with *IDH2*^R140Q^ R/R AML with trisomy 8 in the majority of myeloblasts, with the persistence of trisomy 8 in promyelocytes and mature granulocytes after 4 weeks of enasidenib treatment.^31^ Of note, ivosidenib or enasidenib induces myeloid differentiation and trilineage hematopoietic recovery in *IDH1/2*mt R/R AML without an intercurrent period of bone marrow aplasia or hypoplasia, which is frequently seen during treatment with cytotoxic chemotherapeutic agents, consistent with differentiation as the mechanism of action.^29–31^ This is corroborated by molecular data showing that in many *IDH2*mt R/R AML patients achieving a complete remission (CR) on enasidenib, the *IDH2*mt variant allelic frequencies (VAFs) were unchanged between pre-therapy leukemic cells and neutrophils at the time of CR, consistent with the differentiation of *IDH2*mt leukemia cells into mature neutrophils.^62^ Of note, some patients do achieve a decrease in *IDH2*mt VAF, and these patients have the most durable responses.^30^ This was further confirmed by functional assays, wherein these R/R AML patients in CR on enasidenib had *IDH2*mt-differentiated leukemic neutrophils with intact phagocytic activity, consistent with the restoration of normal granulocyte function.^62^ In additional translational analyses in patient samples, flow cytometry–based immunophenotyping analyses revealed that enasidenib treatment promoted differentiation of not only *IDH2*mt cells, but also *IDH2*wt cells into mature cells.^63^ The differentiation effect of enasidenib treatment on *IDH2*wt cells probably occurs via the abrogation of paracrine *D*-2-HG inhibition on differentiation of *IDH2*wt cells. This evidence of differentiation induction by mtIDH1/2 inhibitors can also be related to decreased DNA hypermethylation after such therapies. Genome-wide methylation is reduced in *IDH1/2*mt AML cells after patients have been treated with mtIDH1/2 inhibitors, but a gene expression profile associated with AML stemness persisted, suggesting that a complete reversal of the mtIDH1/2-induced DNA hypermethylation is not necessary for a clinical response.^54^ In addition to these data, a longitudinal study of patients with *IDH1*mt cholangiocarcinoma that was treated with ivosidenib was performed. Before-treatment and on-treatment biopsies were compared, revealing that mtIDH1 inhibition induced a more differentiated morphology of cholangiocarcinoma cells on H&E staining and the expression of hepatocyte lineage markers *HNF-4α*, *FOXA1* (*HNF-3α*), *FOXA2* (*HNF-3β*), and *PPARA*. These signs of differentiation were associated with a better clinical response to ivosidenib and longer progression-free survival.^64^ Translational data from *IDH1/2*mt glioma treated with mtIDH1/2 inhibitors are not available, probably due to the inherent difficulties of obtaining multiple tumor samples over time in glioma patients.

### Induction of Differentiation by mtIDH1/2 Inhibitors in Patients

In addition, there is supporting evidence for the induction of differentiation by mtIDH1/2 inhibitors from the clinical characteristics of these agents in the treatment of *IDH1/2*mt AML. The most tangible clinical proof of this hypothesis is that ivosidenib and enasidenib can induce a differentiation syndrome in ~20% of patients with *IDH1/2*mt AML, which can even be fatal if left untreated. This adverse effect is called IDH-inhibitor-associated differentiation syndrome (IDH-DS) and is a boxed warning for these drugs. Patients and clinicians should therefore be alert for fever, dyspnea, hypoxia, pulmonary infiltrates, pleural or pericardial effusions, rapid weight gain or peripheral edema, hypotension, lymphadenopathy, bone pain, and hepatic, renal, or multiorgan dysfunction. IDH-DS has many clinical similarities to the differentiation syndrome, or retinoic acid syndrome, that is observed during differentiation therapy with all-trans retinoic acid (ATRA) and arsenic trioxide for acute promyelocytic leukemia (APL).^65^ Once IDH-DS is suspected, corticosteroid therapy and hemodynamic monitoring should be initiated until symptom resolution.^66,67^

Ivosidenib and enasidenib monotherapy lead to a CR in 19–30% and to any response (CR with or without hematologic recovery, partial remission, or a morphologic leukemia-free state) in 40–55% of patients with *IDH1/2*mt AML, with the highest CR and response rates being achieved in ND AML and the lowest rates in R/R AML (see Table 1).^28–31^ Even higher response rates have been observed when mtIDH1/2 inhibitors were combined with intensive chemotherapy or azacitidine in *IDH1/2*mt ND AML,^37–39^ but to be able to isolate the differentiation effects of mtIDH1/2 inhibition we will focus on studies with mtIDH1/2 monotherapy. Among transfusion-dependent patients, ivosidenib and enasidenib facilitated transfusion independence for red blood cells and/or platelets in ~40% of patients.^28–30^ These transfusion independence rates are considerably higher than the CR rate which supports that the mechanism of action of mtIDH1/2 inhibitors relies more on the induction of differentiation rather than cytotoxicity. This is also emphasized by the finding that more than half of the patients who had stable disease after 3 months of treatment, but who did not achieve a reduction in blast counts, reached red blood cell transfusion independence,^30^ whereas transfusion independence is rarely reached without the achievement of a response in other therapies for AML.

**Table 1.** Clinical Efficacy of mtIDH1/2 Inhibitors in Prospective Clinical Trials in Patients With *IDH1/2*mt Cancer.

Treatment	Patient Population	SD Rate (%)	OR Rate (%)	CR Rate (%)	Ref.
mtIDH1 inhibitors					
Ivosidenib monotherapy	*IDH1*mt ND AML		55	30	Roboz et al.^28^
Ivosidenib with azacitidine	*IDH1*mt ND AML		78	61	DiNardo et al.^37^
Ivosidenib with 7+3	*IDH1*mt ND AML		87	68	Stein et al.^38^
Ivosidenib monotherapy	*IDH1*mt R/R AML		42	22	DiNardo et al.^29^
BAY1436032 monotherapy	*IDH1*mt AML		15	4	Heuser et al.^33^
Ivosidenib monotherapy	*IDH1*mt cholangiocarcinoma	51	2	0	Abou-Alfa et al.^26^
Ivosidenib monotherapy	*IDH1*mt glioma	67	3	0	Mellinghoff et al.^34^
Ivosidenib monotherapy	*IDH1*mt chondrosarcoma	52	0	0	Tap et al.^35^
mtIDH2 inhibitor					
Enasidenib with azacitidine	*IDH2*mt ND AML		74	54	DiNardo et al.^39^
Enasidenib with 7+3	*IDH2*mt ND AML		87	55	Stein et al.^38^
Enasidenib monotherapy	*IDH2*mt R/R AML		40	19	Stein et al.^30^
Enasidenib monotherapy	*IDH2*mt MDS		53	0	Stein et al.^36^
Vorasidenib monotherapy	*IDH1/2*mt glioma	73	18	0	Mellinghoff et al.^32^
Other agents					
Azacitidine with venetoclax	*IDH1/2*mt ND AML, ineligible for 7+3			75	DiNardo et al.^68^
Venetoclax with HMA	*IDH1/2*mt ND AML, ineligible for 7+3			86	Pollyea et al.^69^
Venetoclax with HMA/LDAC	*IDH1*mt ND AML, ineligible for 7+3			82	DiNardo et al.^70^
Venetoclax with HMA/LDAC	*IDH2*mt ND AML, ineligible for 7+3			100	DiNardo et al.^70^
Venetoclax with LDAC	*IDH1/2*mt ND AML, ineligible for 7+3			57	Wei et al.^71^
Venetoclax monotherapy	*IDH1/2*mt R/R AML		50	33	Konopleva et al.^72^

Abbreviations: SD, stable disease; OR, overall response; CR, complete remission (for hematologic malignancies) or complete response (for solid tumors); ND, newly diagnosed; AML, acute myeloid leukemia; 7+3, intensive chemotherapy with 7 days of cytarabine and 3 days of an anthracycline; R/R, relapsed or refractory; MDS, myelodysplastic syndromes; HMA, hypomethylating agent/DNA methyltransferase inhibitor, that is, azacitidine or decitabine; LDAC, low-dose cytarabine.

In addition, the median time to first response (~2 months) and the median time to best response (~3–4 months) of ivosidenib and enasidenib are more similar to the response patterns of hypomethylating therapy with decitabine, azacitidine, or low-dose cytarabine than with intensive chemotherapy.^29–31,39,73,74^ This similarity may be relevant because at low doses, hypomethylation agents also mainly work via the induction of differentiation and only limitedly via cytotoxic effects and can require 4–6 months to induce a response.^74^ Furthermore, *IDH1/2*mt VAFs were studied before and during treatment with mtIDH1/2 inhibitors. Responses to mtIDH1/2 inhibitors were observed in patients with clonal *IDH1/2*mt and patients with subclonal *IDH1/2*mt, suggesting that ancestral or non-ancestral *IDH1/2*mt clones are both amenable to respond to mtIDH1/2 inhibitors.^33,62,75^ This is relevant because in ancestral *IDH1/2*mt clones the *IDH1/2*mt is likely present in the CSC compartment, whereas in non-ancestral *IDH1/2*mt clones the *IDH1/2*mt may not be present in the CSC compartment.^18^ Ivosidenib and enasidenib induced an *IDH1/2*mt molecular remission, defined as an *IDH1/2*mt VAF below the limit of detection (0.02–0.04%), in ~10–30% of patients, and all of them had a hematologic response, often a CR (~80–100%).^28–30^ As expected, patients with an *IDH1/2*mt molecular remission were more likely to achieve a CR and a significantly longer overall survival than patients without a molecular remission (15 months vs 10 months for ivosidenib and 23 months vs 9 months for enasidenib), suggesting that *IDH1/2*mt clearance from all AML cells, including the CSC compartment, is necessary for a deep and durable remission.^29,30,54^

With respect to the pharmacodynamics of mtIDH1/2 inhibitors, variable efficacies with respect to plasma *D*-2-HG level reductions have been described, but the relationship is unclear between the extent of the decrease in plasma *D*-2-HG levels and the likelihood of a clinical response. For example, ivosidenib generally achieves a near-complete suppression of plasma *D*-2-HG levels in *IDH1*mt R/R AML patients, but many patients with such near-complete *D*-2-HG reductions do not respond to ivosidenib. More specifically, plasma *D*-2-HG was reduced by a median of 95% in patients who achieved a response and an almost equal median of 94% in those who did not achieve a response.^29^ On the contrary, this disconcordance between suppression of plasma *D*-2-HG levels and clinical benefit might be disease-specific to AML because in cholangiocarcinoma, treatment duration, which may be a surrogate for clinical efficacy, appeared to be associated with suppressed plasma *D*-2-HG levels after one cycle of ivosidenib treatment.^27^ In the context of enasidenib, a more potent reduction of plasma *D*-2-HG levels was achieved in *IDH2*^R140Q^ than in *IDH2*^R172K^ R/R AML patients (median −93% vs median −47%).^30,31^ In patients with *IDH2*^R140Q^ R/R AML, the level of plasma *D*-2-HG reduction by enasidenib was not associated with a clinical response, with plasma *D*-2-HG reductions of −94%, −95%, and −90% in patients with a CR, non-CR response, or no response, respectively. In contrast, plasma *D*-2-HG suppression by enasidenib was associated with the clinical response in *IDH2*^R172K^ R/R AML patients, with plasma *D*-2-HG reductions of −82%, −44%, and −38% in the abovementioned categories, respectively.^30^ These *D*-2-HG reductions are not complete, not even in patients achieving a CR, because of background *D*-2-HG production in non-malignant cells by other enzymes than mtIDH1/2. In addition, these data show that near-complete abrogation of *D*-2-HG production by *IDH2*mt AML cells is not always sufficient to induce a CR. This suggests that *IDH1/2*mt AML can develop resistance mechanisms that confer *D*-2-HG independence or that *D*-2-HG production is not homogeneously stopped in all malignant cells within *IDH1/2*mt AML with residual *D*-2-HG production still occurring in the CSC compartment.

## Resistance Mechanisms to mtIDH1/2 Inhibitors

Several mechanisms of primary and secondary resistance to mtIDH1/2 inhibitors have been described. In primary resistance, a patient fails to respond to a therapy. In secondary resistance, relapse occurs after an earlier response to a therapy. In both cases, the CSC compartment may play a crucial role.

With respect to primary resistance to mtIDH1/2 inhibitors, an universal mechanism across various types of *IDH1/2*mt cancers has not yet been described. In *IDH1/2*mt AML, baseline co-mutations in genes of the receptor tyrosine kinase (RTK) pathway (*NRAS, FLT3*, and *PTPN11)* and hematopoietic differentiation transcription factors (*RUNX1, CEBPA*, and *GATA2*) are negatively associated with the occurrence of a response to mtIDH1/2 inhibitors.^30,62,75^ These findings can be partially extended to *IDH1/2*mt cholangiocarcinoma, in which gene signatures associated with activated RTK and PI3K/AKT-activity are associated with early progression upon treatment with ivosidenib.^64^ In glioma, the presence of genetic alterations in cell cycle pathway genes was associated with a shorter progression-free survival.^34^ In addition to these genetic hallmarks of mtIDH1/2-inhibitor primary resistance, leukemia stemness is associated with poor responses to mtIDH1/2 inhibitors in *IDH1/2*mt AML. In primary samples of patients with *IDH1/2*mt AML treated with mtIDH1/2 inhibitors, resistance to mtIDH1/2 inhibitors was more frequently observed in patients with versus patients without a hypermethylated genotype with a gene expression profile associated with AML stemness. In addition, a genetic CSC risk score, the LSC17,^76^ was a better predictor for response to mtIDH1/2 inhibition than the established and aforementioned molecular risk factors such as cytogenetics, *RUNX1* mutation status, or *RAS/RTK* mutation status.^54^

With respect to secondary resistance to mtIDH1/2 inhibitors, several mechanisms have been described, and they may be more universal across the various types of *IDH1/2*mt cancers. First, there is the emergence of downstream mutations, such as *TET2, BCOR*, differentiation genes (*RUNX1, GATA2, CEPBA*), and RTK genes (*RAS, FLT3, PTPN11, KIT*).^54,63,75,77^ Second, *IDH1/2*mt patients who relapse on mtIDH1/2 inhibitor therapy may do so because of *D*-2-HG-restoring mutations.^54,63,75,78,79^ Restoration of *D*-2-HG production during mtIDH1 or mtIDH2 inhibition can result either from a second-site mutation in the inhibited IDH1/2 homolog that prevents binding of the mtIDH1/2 inhibitor or from a process termed isoform switching or homolog switching, in which an *IDH2*mt emerges during mtIDH1 inhibition or vice versa.^54,63,75,78,79^ In patients with *IDH1*mt R/R AML, 35% of patients who achieved a response on enasidenib developed such a *D*-2-HG-restoring mutation, with second-site mutations and homolog switching occurring in equal proportions of patients (~20%). Second-site mutations, such as *IDH1*^S280F^, *IDH1*^R119P^, *IDH2*^Q316E^, and *IDH2*^I319M^,^54,75,78^ occur outside of the catalytically active site of the IDH1/2 enzymes and are modeled to directly or indirectly prevent the binding of allosteric mtIDH1/2 inhibitors such as ivosidenib and enasidenib.^75^ Among relapsing *IDH1*mt R/R AML patients who exhibited isoform switching, most developed an *IDH2*mt that was not yet detected at baseline, either in the same clone that was previously *IDH1*mt or in a separate clone.^75^ A smaller proportion of patients had two co-existing clones, one *IDH1*mt and one *IDH2*mt, with the relapse driven by the *IDH2*mt clone during mtIDH1 inhibition.^75^ A final mechanism of acquired mtIDH1 inhibitor resistance was described in *IDH1*mt cholangiocarcinoma in which an *IDH1*^R132C^ mutation converted to an *IDH1*^R132F^ mutation, suggesting that the phenylalanine instead of the cysteine at residue 132 confers resistance to ivosidenib.^77^

Currently, second-generation mtIDH1/2 inhibitors are in development, which may decrease the secondary resistance rates to mtIDH1/2 inhibition. Vorasidenib inhibits both mtIDH1 and mtIDH2 at nanomolar concentrations^80^ and is currently under investigation for *IDH1/2*mt glioma.^32^ Its dual mtIDH1/2 inhibition will likely reduce the possibility for *IDH1/2*mt cells to escape mtIDH1/2 inhibition via isoform switching. In addition, a novel type of inhibitor that is in preclinical testing does not target the allosteric site, but instead binds the active site of mtIDH1,^81^ thereby decreasing the possibility for *IDH1/2*mt to restore their *D*-2-HG production via a second-site mutation at the allosteric site.

## Combination Therapies With mtIDH1/2 Inhibitors

Another therapeutic strategy that may increase the clinical benefit of mtIDH1/2 inhibitors or decrease the risk of resistance is combining these drugs with other anti-cancer agents. For example, ivosidenib and enasidenib have been combined with intensive chemotherapy or azacitidine in *IDH1/2*mt ND AML,^37–39^ and these strategies achieved higher response rates than ivosidenib or enasidenib monotherapy (see Table 1). This supports the notion that the best clinical outcomes in patients with *IDH1/2*mt cancer may be achieved by a combination of an mtIDH1/2 inhibitor with another targeted agent or chemotherapy.^37–39^ Although preclinical evidence suggested that mtIDH1/2 inhibitors decrease the effect of cytotoxic therapy in *IDH1/2*mt cancer cells,^82–84^ impressive response rates have been achieved with ivosidenib or enasidenib combined with induction and consolidation chemotherapy in patients with *IDH1/2*mt ND AML.^38^ However, this study was not randomized and thus cannot confirm or contradict the aforementioned preclinical warnings against combining mtIDH1/2 inhibitors with cytotoxic therapy. Whether or not adding mtIDH1/2 inhibitors to intensive chemotherapy has clinical benefit in patients with *IDH1/2*mt ND AML is currently being studied in a randomized clinical trial.^38^

Combining azacitidine and enasidenib in vitro results in greater reductions in DNA methylation and enhanced EPO-induced erythroid differentiation in an erythroleukemia cell line overexpressing mtIDH2, compared with azacitidine or enasidenib alone. This is consistent with a model wherein enasidenib-induced reactivation of TET DNA demethylase enzymes contributes to azacitidine-induced inhibition of DNA methyltransferase enzymes.^85^ In agreement with these preclinical results, a high CR rate of 61% was achieved with ivosidenib plus azacitidine in patients with *IDH1*mt ND AML ineligible for intensive chemotherapy in a non-randomized clinical trial.^37^ Moreover, in a randomized clinical trial, the CR rate was significantly higher in the enasidenib plus azacitidine group compared with the azacitidine-only group (54% vs 12%).^39^

Another potential powerful combination is that of an mtIDH1/2 inhibitor and the BCL2 inhibitor venetoclax. *IDH1/2*mt cancers are sensitive to BCL2 inhibition because *D*-2-HG accumulation induces BCL2 dependence via inhibition of cytochrome c oxidase, also known as complex IV of the electron transport chain. This effect lowered the threshold to trigger mitochondrial apoptosis upon BCL2 inhibition by venetoclax and rendered *IDH1/2*mt AML cells 13-fold more sensitive to venetoclax compared with *IDH1/2*wt AML cells.^86^ This observation is corroborated by clinical data, in which venetoclax achieved a modest 33% CR rate when administered as monotherapy to *IDH1/2*mt R/R AML patients,^72^ but high and durable CR rates (57–100%) when administered in combination with azacitidine, decitabine, or low-dose cytarabine in patients with *IDH1/2*mt ND AML who were ineligible for intensive chemotherapy (see Table 1).^68–71^ However, the finding that *D-*2-HG increases BCL2 dependency may also impose a limitation on combining mtIDH1/2 inhibitors and venetoclax. Suppression of *D*-2-HG production by an mtIDH1/2 inhibitor may decrease the BCL2 dependency of *IDH1/2*mt cancer cells and instead facilitate resistance against venetoclax. On the contrary, mtIDH1/2 inhibitor-induced differentiation may further increase venetoclax activity on *IDH1/2*mt cells by lowering the apoptotic threshold, as has been observed with other agents that promote differentiation.^87^ In vitro and in vivo experiments with an erythroleukemia cell line and primary AML samples suggested that enasidenib-induced differentiation further sensitizes *IDH2*mt AML cells to venetoclax, but that venetoclax might be antagonized by enasidenib when it fails to induce differentiation when administered as enasidenib monotherapy. A conundrum here is that in all three primary AML samples studied as mouse xenografts, the combination of enasidenib and venetoclax induced more differentiation as measured by CD15 expression than enasidenib monotherapy alone, even when enasidenib seemed to antagonize the antiproliferative effect of venetoclax.^87^ Clinical trials on the combination of an mtIDH1/2 inhibitor and venetoclax are currently ongoing.

## Other Differentiation Strategies Directly or Indirectly Targeting mtIDH1/2

In addition to mtIDH1/2 inhibitors, other differentiation agents may be used to induce differentiation of *IDH1/2*mt AML cells. For example, *IDH1/2*mt induces H3K4 trimethylation of the *CEBPA* promoter and thereby increases the expression of this transcription factor in AML cells. In the context of normal hematopoiesis and AML, CEBPα expression is associated with the granulocyte-monocytic progenitor step and *IDH1/2*mt AML cells are therefore locked in this stage. This block in differentiation was associated with a gene expression signature that was enriched for genes responsive to treatment with ATRA, analogous to promyelocytic leukemia/retinoic acid receptor α (PML/RARα)-driven APL. This was supported by experiments in primary *IDH1/2*mt AML mouse xenografts, in which ATRA-induced differentiation of AML blasts to more differentiated granulomonocytic cells was achieved in a similar fashion as APL, but not primary *IDH1/2*wt AML xenografts.^88^

As mentioned above, mtIDH1/2 inhibitors can induce transfusion independence for red blood cells and/or platelets in *IDH1/2*mt AML patients.^28–30^ An interesting observation is that enasidenib can also induce hematopoietic differentiation independently of wtIDH2 or mtIDH2. In IDH2-deficient hematopoietic-progenitor cells, enasidenib but no other mtIDH1/2 inhibitors induced differentiation to mature erythrocytes. This process was mediated by accumulation of protoporphyrin IX, the direct precursor of heme, by virtue of off-target inhibition of enasidenib on the ATP-binding cassette subfamily G member 2 (ABCG2), a transporter highly expressed in erythroid progenitors which is responsible for efflux of protoporphyrin IX. Therefore, enasidenib may be a promising therapeutic agent for improvement of anemia in a wide array of clinical contexts outside of *IDH1/2*mt cancers.^89^ This mechanism is supplementary to the mtIDH1/2-inhibitor-induced restoration of the abovementioned deficiency of succinyl-CoA in *IDH1/2*mt myeloid progenitor cells, a critical component for heme biosynthesis.^51^

## Concluding Remarks and Future Perspectives

*IDH1/2*mt are attractive therapeutic targets for various reasons, but most prominently because they are early events in oncogenesis and are therefore present in a large proportion of cancer cells, including the CSC compartment. These mutations induce a block in differentiation early in the maturation of cells in glial, cartilaginous, biliary, and myeloid tissues, and mtIDH1/2 inhibitors potently reverse the metabolic effects of mtIDH1/2 and release differentiation of *IDH1/2*mt cells into more mature cells. Because the mechanism of action is different and/or complementary to that of cytotoxic agents, other hypomethylating agents and other targeted agents such as venetoclax, we envisage that mtIDH1/2 inhibitors can be most efficaciously used in combination with these agents and that multiple classes of drugs can collaborate in the optimal eradication of the CSC compartment and, therefore, long-term remissions for *IDH1/2*mt cancer patients.
